# Promising approaches for simultaneous enhancement of medicinally significant benzylisoquinoline alkaloids in opium poppy

**DOI:** 10.3389/fpls.2024.1377318

**Published:** 2024-04-02

**Authors:** Zahra Aghaali, Mohammad Reza Naghavi, Meisam Zargar

**Affiliations:** ^1^ Department of Genetics and Plant Breeding, Faculty of Agriculture, Tarbiat Modares University, Tehran, Iran; ^2^ Division of Plant Biotechnology, Department of Agronomy and Plant Breeding, College of Agricultural and Natural Resources, University of Tehran, Karaj, Iran; ^3^ Department of Agrobiotechnology, Agrarian Technological Institute, Peoples' Friendship University of Russia (RUDN) University, Moscow, Russia

**Keywords:** autopolyploidy, CRISPR/Cas9, elicitor, opium poppy, PsWRKY, synthetic metabolon

## Abstract

Benzylisoquinoline alkaloids (BIAs) produced in opium poppy have been evidenced to heal patients suffering from various diseases. They, therefore, hold an integral position in the herbal drug industry. Despite the adoption of several approaches for the large-scale production of BIAs, opium poppy remains the only platform in this purpose. The only disadvantage associated with producing BIAs in the plant is their small quantity. Thus, recruiting strategies that boost their levels is deemed necessary. All the methods which have been employed so far are just able to enhance a maximum of two BIAs. Thus, if these methods are utilized, a sizable amount of time and budget must be spent on the synthesis of all BIAs. Hence, the exploitation of strategies which increase the content of all BIAs at the same time is more commercially effective and time-saving, avoiding the laborious step of resolving the biosynthetic pathway of each compound. Exposure to biotic and abiotic elicitors, development of a synthetic auto-tetraploid, overexpression of a WRKY transcription factor, formation of an artificial metabolon, and suppression of a gene in the shikimate pathway and miRNA are strategies that turn opium poppy into a versatile bioreactor for the concurrent and massive production of BIAs. The last three strategies have never been applied for BIA biosynthetic pathways.

## 1 Introduction

Opium poppy (*Papaver somniferum* L.) is widely known as a medicinally significant plant that produces a sizable number of alkaloids, virtually 80 (Hagel and Facchini, 2013). Among these nitrogen-containing compounds are benzylisoquinoline alkaloids (BIAs) which comprise a diverse group of metabolites in terms of structure and function. Morphine, codeine, thebaine, sanguinarine, papaverine, and noscapine are well-established BIAs occurring mainly in opium poppy. Morphine has been administered for sleep disorders, acute pains, and diarrhea (Hamilton and Baskett, 2000). Codeine has the potential to relieve painful muscle tension and to induce sleep as well (Facchini and Park, 2003). Thebaine has been recognized as a sedative agent (Pathan and Williams, 2012). Sanguinarine is a potent drug that remedies inflammation (Dvorák et al., 2006). The healing power of papaverine against muscle spasms and vasoconstriction has been reported (Desgagne´-Penix and Facchini, 2012). Noscapine has been widely prescribed for severe cough and a wide range of cancers (Mahmoudian and Rahimi-Moghaddam, 2009; Rahmanian-Devin et al., 2021). Interestingly, the inhibitory effect of sanguinarine and papaverine on tumorous cells has also been proven (Desgagne´-Penix and Facchini, 2012; Galadari et al., 2017).

If dropping out sanguinarine, the biosynthesis of BIA is strictly associated with differentiation and the development of specific cell types (Facchini and Bird, 1998; Rezaei et al., 2016a; Rezaei et al., 2016b). Thus, with the exception of sanguinarine, cell suspension culture fails to produce BIAs as they lack differentiation. Although offering accelerated and accessible opportunities to synthesize BIA, synthetic approaches are unable to satisfy the long-term advantage. Given that BIAs possess one or more chiral centers and petroleum-based solvents are being used for the production and purification of intermediates and target products (Wu and Chappell, 2008), the chemical synthesis of BIAs is not straightforward, cost-competitive, and environmentally friendly. Carrying differentiated cells and utilizing sunlight and CO_2_ instead of reactants derived from petroleum, opium poppy is a sole and sustainable option for BIA production. However, their low quantity in poppy plants imposes a barrier to produce them commercially. This prompted us to summarize as well as propose biotechnological strategies beneficial for the concurrent enhancement of the BIA content in opium poppy.

## 2 Biotechnological interventions to increase BIA content in opium poppy

### 2.1 Elicitation

Elicitation has recently emerged as an effective biotechnological approach for stimulating or enhancing plant secondary metabolites. Elicitors are substances of biotic or abiotic origin that trigger plant defense responses and secondary metabolism. This assists the elicited plants to retain their productivity and viability (Jeyasri et al., 2023). Elicitors fall into two categories: abiotic and biotic. Abiotic elicitors can be further divided into physical, chemical, and hormonal stimuli, among which are salinity, drought, UV radiation, heavy metals, nanoparticles, methyl jasmonate (MeJA), and salicylic acid (SA). Signals originated from endophytic fungi or bacteria, pathogenic microorganisms, including lysates and yeast extract, or plants, such as polysaccharides, glycoproteins, and pectin which are counted as biotic elicitors. The biological and non-biological factors have elevated the capacity of opium poppy for producing and accumulating BIAs (**Table 1**).

**Table 1:** Positive impacts of abiotic and biotic elicitors on the benzylisoquinoline alkaloid contents produced in *Papaver somniferum* L.

Species	Origin	Elicitor	Duration	Concentration	Metabolite	Enhancement rate	Reference
*Papaver somniferum* L.	Abiotic	MeJA	12 h	100 μM	Morphine Noscapine Thebaine	1.8 fold 1.6 fold 3.2 fold	Gurkok et al., 2014
		TiO_2_	24 h	120 mg 120 mg	Sanguinarine Thebaine	2.2 fold 3.4 fold	Khodayari et al., 2014
		Drought	5 days	–	Morphine Codeine Noscapine	4.2 fold 1.1 fold 4.2 fold	Szabó et al., 2003
	Biotic	YE	48 h	0.2 mg	Sanguinarine Thebaine	2.7 fold 2.5 fold	Khodayari et al., 2014
		SM1B	72 h	10 ml	Morphine Noscapine Papaverine	1044% 936% 349%	Pandey et al., 2016
		SM10B	72 h	10 ml	Thebaine	718%	Pandey et al., 2016

MeJA, methyl jasmonate; TiO_2_, titanium dioxide; YE, yeast extract; SM1B, Acinetobacter; SM10B, Kocuria sp. –, stress has not concentration.

**Figure 1:**
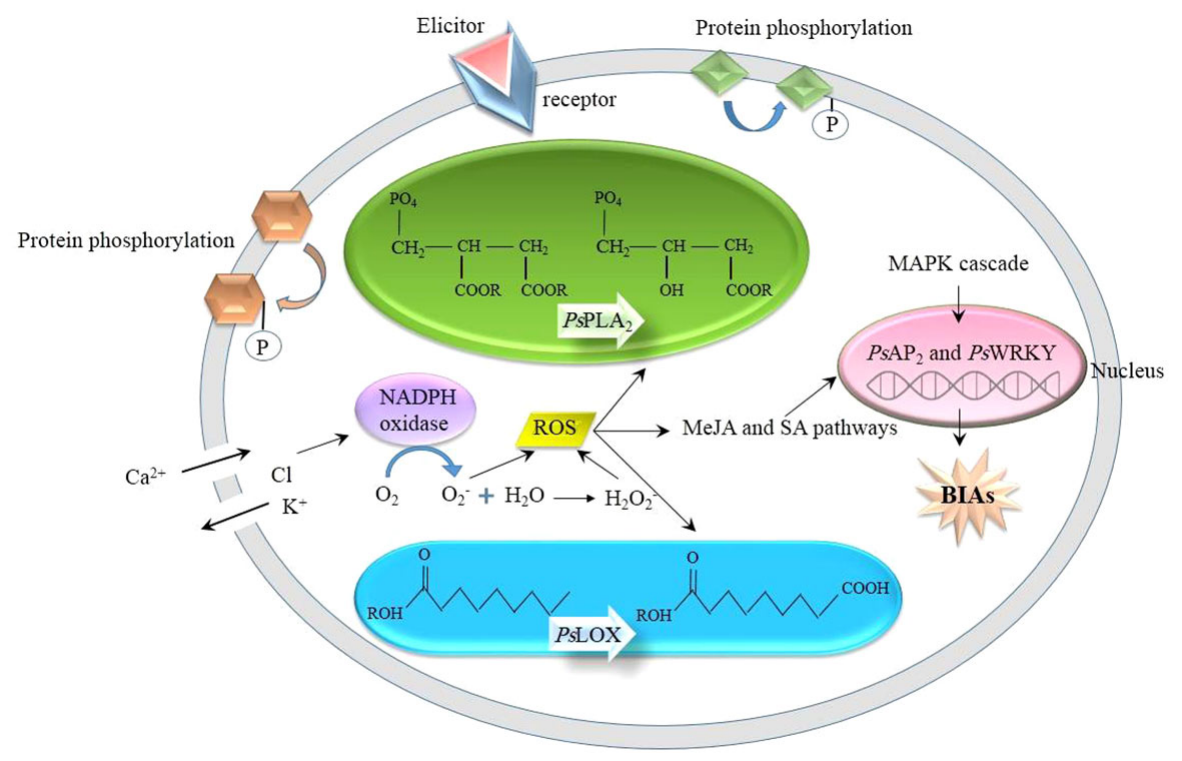
Woven events which are triggered by an external elicitor. BIAs, benzylisoquinoline alkaloids; MAPK; mitogen-activated protein kinase; MeJA, methyl jasmonate; *Ps*LOX, lipoxygenase; *Ps*PLA_2_, phospholipases A_2_; ROS, reactive oxygen species; SA, salicylic acid.

The underlying mechanism whereby an elicitor induces the biosynthesis of BIA in opium poppy is analogous to that of other phytochemical compounds in other plant species. When challenged by abiotic and biotic stimuli, the poppy plants witness a series of intracellular events, ranging from physiological to biochemical (**Figure 1**): The receptor-like kinases (RLKs) and receptor-like proteins (RLPs) anchoring the plasma membrane sense an external elicitor (Liang and Zhang, 2022), initiating the transfer of the signal to the downstream proteins present in the plasma membrane and cytosol, resulting in their reversible phosphorylation. The receptors are ubiquitous in the plant kingdom. Meanwhile, in opium poppy, the activities of phospholipase A_2_ (*Ps*PLA_2_) and lipoxygenase (*Ps*LOX) are increased. *Ps*PLA_2_ hydrolyzes phospholipids, generates fatty acids (Jablonická et al., 2016), and takes a prominent role in MeJA biosynthesis and signaling (Jeyasri et al., 2023), whereas *Ps*LOX is responsible for the stereospecific deoxygenation of polyunsaturated fatty acids, such as linoleic and linolenic acids (Holková et al., 2010). In addition to that, similar to other plant species, ions are channeled into the inside and outside of the cell, i.e., Ca^2+^ and proton influx as well as Cl^-^ and K^+^ efflux, resulting in cytosol acidification as well as apoplast alkalization (Humbal and Pathak, 2023). The deficiency of K^+^ stimulates the expression of NADPH oxidase which catalyzes the production of reactive oxygen species (ROS), such as O_2_^-^ and H_2_O_2_ (Hajiboland, 2012). Following ROS burst, the synthesis of defense chemicals, including flavonoids, tannins, and phytoalexins, is enhanced. In addition, ROS trigger MeJA and SA signaling. With the MeJA signaling pathway, *Ps*AP2/ERF and *Ps*WRKY transcription factors are upregulated in opium poppy, overexpressing various stress-responsive genes, such as BIAs (Mishra et al., 2013; Mishra et al., 2015). Additionally, the mitogen-activated protein kinase (MAPK) pathway is also activated as the other plant species. Through the MAP kinase cascade, MAP kinase kinases (MAPKKK) phosphorylate MAP kinase kinases (MAPKK) followed by the phosphorylation of MAP kinases (MAPK) by MAPKK. The phosphorylated MAPKs are directed to the nucleus in which they phosphorylate group 1 WRKY transcription factors containing two WRKY domains, WRKYGQK, and a zinc finger motif in the N- and C- terminals, respectively (Ishihama and Yoshioka, 2012). In opium poppy, *Ps*WRKY is characterized as the group 1 WRKY (Agarwal et al., 2016), participating in response to wounding, cold, dehydration, and salt stresses (Mishra et al., 2013). *Ps*WRKY is supposed to regulate the BIA pathway (Mishra et al., 2013) so that its activation by phosphorylation promotes the expression of the genes involved in BIA biosynthetic pathways and ultimately increases BIA concentration.

### 2.2 Induction of autopolyploidy

**Figure 2:**
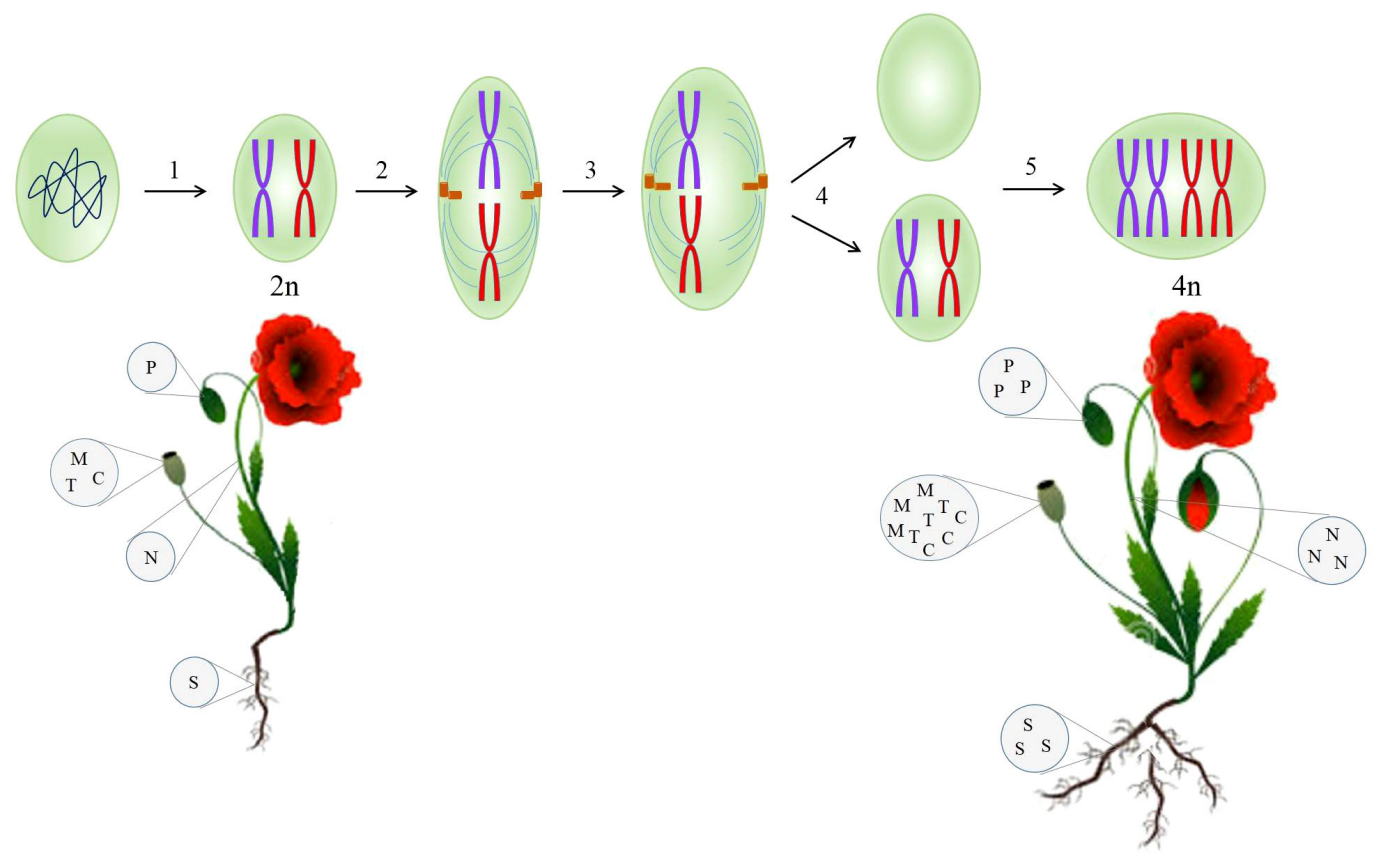
Synthetic autotetraploid developed from a diploid cell through disruption in the mitosis cycle mediated by colchicine, enhancing the biomass and secondary metabolites contents in the resulting plant. 1, prophase; 2, metaphase; 3, anaphase; 4, telophase; 5, S-phase. C, codeine; M, morphine; N, noscapine; P, papaverine; S, sanguinarine; T, thebaine.

Following at least two cycles of whole-genome doubling, polyploid plants are arisen. Genome duplication encourages adaptability, sustainability, tolerance toward biotic and abiotic stresses, growth, and diversity of plants during their historical evolution (Madani et al., 2021). Polyploidization has occurred by either allopolyploidy or autopolyploidy. Allopolyploid organisms arise from hybridization between two genetically divergent species and then the duplication of chromosomes (Yang et al., 2011). On the other hand, autopolyploids are derived from a disturbance in meiotic segregation where chromosomes within a species are multiplied without the subsequent cell division (**Figure 2**) (Yang et al., 2011). Autopolyploidy can also be induced by the application of chemical and physical agents. The well-known chemicals inhibiting the mitotic cell cycle are colchicine, oryzalin, nitroxide, and dinitroanilines. X-ray, UV radiation, and gamma ray are recognized as physical mutagens (Salma et al., 2017).

Naturally, opium poppy underwent two independent genome doubling events, accounting for 7.8 million years ago and a preceding one (Guo et al., 2018). The presence of duplicated genes whose enzymes participate in BIA metabolism proves this evolutionary history in opium poppy (Hu et al., 2018). To take advantage of this natural incident, a synthetic autopolyploid opium poppy was created using exposure to colchicine (Mishra et al., 2010). As a result of doubling chromosome sets, all the genes involved in BIA pathway showed the higher expression contrary to the diploid counterparts (Mishra et al., 2010). It has been documented that there is a positive correlation between polyploidy and an increase in genetic materials indispensable for transcription, such as DNA template and transcription factors. That is why autopolyploidy enhances gene expression (Mishra et al., 2010; Madani et al., 2021) and ultimately the secondary metabolites concentration (**Figure 2**). A dramatic increase in morphine and noscapine contents (50%) was observed in artificially synthesized autopolyploid opium poppy (Mishra et al., 2010), which can be attributed to the elevated enzymatic and metabolic activities (Dhawan and Lavania, 1996).

## 3 Future strategies to enhance BIA content in opium poppy

### 3.1 Overexpression of PsWRKY

WRKY proteins are principal contributors to plant immunity, metabolism, growth, and development. They transcriptionally regulate genes involved in several biological processes, including biotic and abiotic stress responses (Lee et al., 2018), production of elicitor-activating chemicals, phytohormone synthesis (Schluttenhofer and Yuan, 2015), and senescence (Rushton et al., 2010; Cai et al., 2014). Members of the WRKY family are discriminated from the other via one or more copies of a conserved WRKY domain (WRKYGXK) required for binding to DNA at the N-terminal and a zinc finger motif (CX4-8-C-X22-28-H-X1-2-H/C) at the C-terminus (Eulgem et al., 2000). It has been proven that the WRKY transcription factors function as the transcriptional regulator of the genes responsible for alkaloid biosynthesis (Mishra et al., 2013). In terms of BIAs occurring in opium poppy, *Ps*WRKY assumes the responsibility of expressing virtually all genes involved in these pathways (Kawano et al., 2012; Mishra et al., 2013) as the W-box element (TTGACC/T) interacting with the WRKY domain has been recognized in the promoter regions of these genes (Winzer et al., 2012; Agarwal et al., 2016). Moreover, a close association between the gene transcript encoding *Ps*WRKY and tissues synthetizing and accumulating BIAs was reported in opium poppy (Mishra et al., 2013). The other transcription factor isolated from opium poppy is MYB (Kakeshpour et al., 2015). MYB protein is a key transcriptional regulator of genes involved in the monoterpenoid indole alkaloid (MIA) biosynthesis (Labanca et al., 2018). It has been evidenced that MYB motifs are only present in the promoter regions of 10 genes responsible for noscapine synthesis (Winzer et al., 2012). Thus, this TF lacks the capability of comprehensively regulating BIA production, highlighting the fact that the *Ps*WRKY overexpression should be prioritized in this regard.

**Figure 3:**
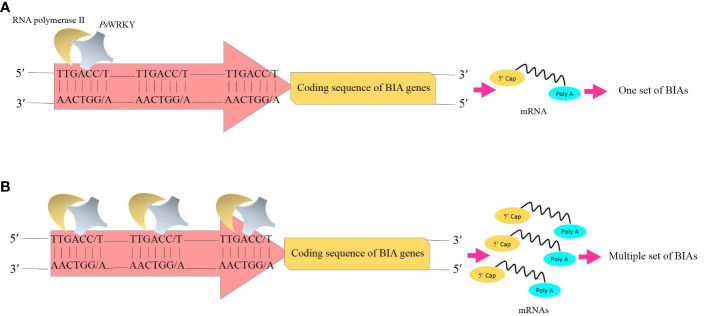
Transcription of the genes whose enzymes are involved in the BIA biosynthetic pathways. **(A)** Binding of one copy of transcription factor *Ps*WRKY to one W-box (TTGACC/T) in the promoter and subsequently the synthesis of one set of BIAs. **(B)** Overproduction of BIAs results from the overexpression of *Ps*WRKY and multiple occupancy of W-boxes. BIAs, benzylisoquinoline alkaloids; *Ps*WRKY, *Papaver somniferum* WRKY transcription factor.

Transcription factors are known as one of the feasible targets for global manipulation of metabolic pathways. In California poppy, a plant species belonging to the *Papaver* genus with the BIA production capability, BIA biosynthesis was substantially enhanced as a result of the overexpression of AtWRKY1 isolated from *Arabidopsis thaliana* (Apuya et al., 2008). Accordingly, given the fact that *Ps*WRKY is a comprehensive activator of the BIA biosynthetic genes, upregulation of the transcription factor is speculated to result in the synchronized accumulation of BIAs in opium poppy through the proposed sequential processes: The *Ps*WRKY protein scans DNA to find the target site (W-box) that shows high affinity for the transcription factor. Following the recognition of the W-box, *Ps*WRKY specifically interacts with it. The other *Ps*WRKYs likewise head the same manner and bind with the other W-boxes. After a while, multiple W-boxes scattered in the promoter regions of the BIA biosynthetic genes (Winzer et al., 2012; Agarwal et al., 2016) are occupied by *Ps*WRKYs. They coordinately initiate the transcription of the genes. Subsequently, several transcripts are produced at the same time. With an enhancement in transcripts, enzymes translated from them are increased as well, resulting in an increase in the number of reactions mediated by these enzymes. Finally, the BIA amount is boosted (**Figure 3**).

### 3.2 Construction of a metabolon

Metabolon (or fusion protein) refers to a perpetual or transient complex of enzymes, in which an intermediate produced by one enzyme is directly transferred to the subsequent enzyme without diffusing into the bulk phase of the cell. Aligning active sites of sequential enzymes protects the cell against toxic intermediates, prevents the consumption of intermediates by competitive pathways, and accelerates the reaction rate (Sweetlove and Fernie, 2018). This phenomenon has been evidenced in primary and secondary metabolisms (Obata, 2019). In opium poppy, the biosynthetic pathway of morphine and its derivatives, codeine and thebaine, enjoys a fusion between a cytochrome P450 (CYP) and an oxidoreductase. The CYP enzyme shoulders the conversion of (*S*)-reticuline to 1,2-dehydroreticuline, while another enzyme, oxidoreductase, is in charge of forming (*R*)-reticuline from 1,2-dehydroreticuline (Farrow et al., 2015; Winzer et al., 2015).

**Figure 4:**
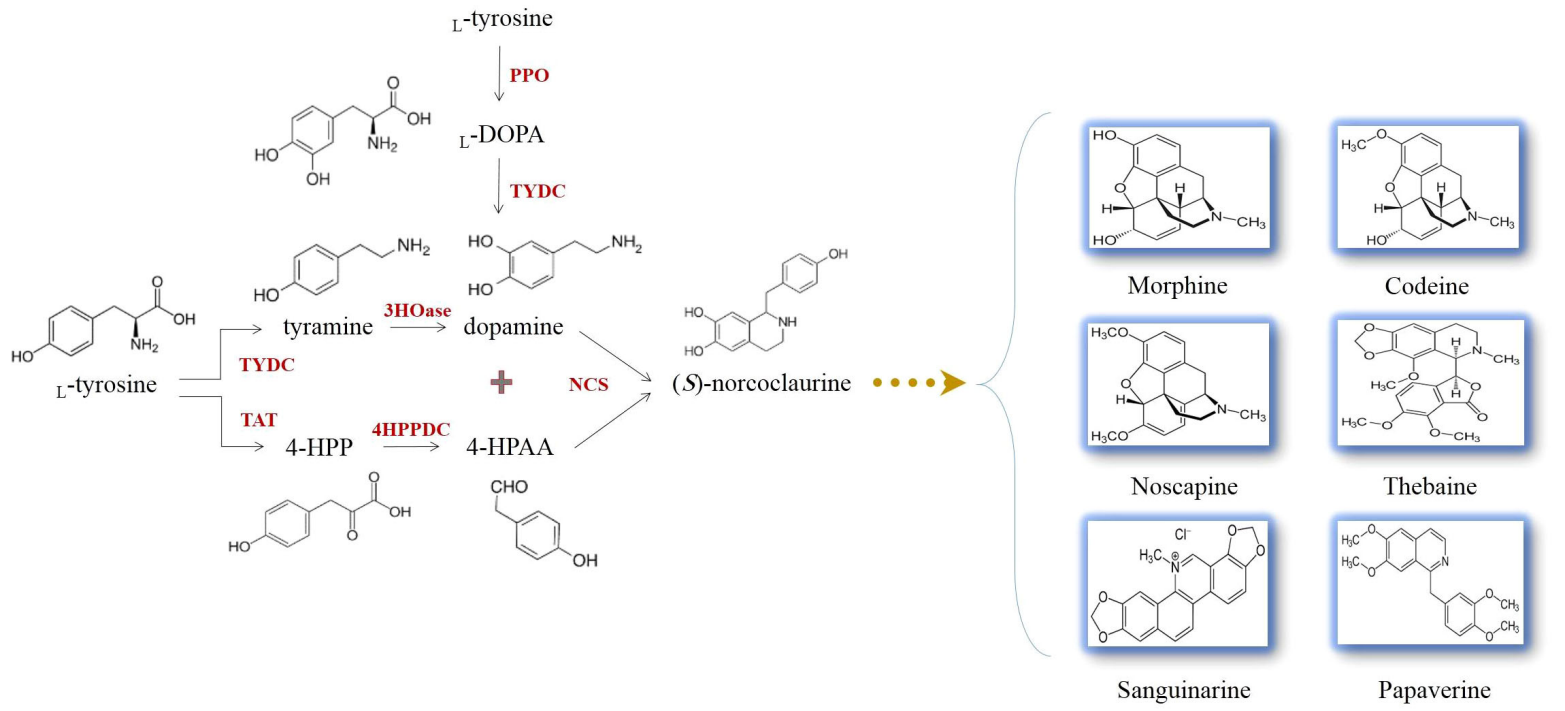
The production of (S)-norcoclaurine allows the biosynthesis of L-tyrosine-derived benzylisoquinoline alkaloids occurring in opium poppy. L-DOPA, 3,4-dihydroxy-L-phenylalanine; 3HOase, L-tyrosine/tyramine 3-hydroxylase; 4-HPAA, 4-hydroxyphenylacetaldehyde; 4-HPP, 4-hydroxyphenylpyruvate; 4HPPDC, 4-hydroxyphenylpuruvate decarboxylase; NCS, norcoclaurine synthase; PPO, polyphenol oxidase; TAT, L-tyrosine aminotransferase; TYDC, L-tyrosine/L-DOPA decarboxylase.

It is evidenced that all BIAs in opium poppy are originated from a common precursor, (*S*)-norcoclaurine (**Figure 4**). (*S*)-norcoclaurine is yielded by the condensation of dopamine and 4-hydroxyphenylacetaldehyde (4HPAA) as donors of amine and aldehyde moieties, respectively (Beaudoin and Facchini, 2014) (**Figure 4**). This reaction which is mediated by norcoclaurine synthase (NCS) allows entry into BIA metabolism (Samanani et al., 2004). With the activities of an aminotransferase coupled with an uncharacterized 4-hydroxyphenylpyruvate decarboxylase (4HPPDC), _L_-tyrosine converts to 4HPAA in the consecutive reactions (**Figure 4**) (Lee and Facchini, 2011). The _L_-tyrosine decarboxylation performed by _L_-tyrosine/_L_-DOPA decarboxylase (TYDC) generates tyramine which subsequently undergoes 3-hydroxylation, resulting in dopamine synthesis (Facchini and De Luca, 1994). The enzyme catalyzing the hydroxylation reaction has not yet been identified in plants (**Figure 4**). Dopamine has been found to be synthesized from an alternative route. The hydroxylation of _L_-tyrosine by polyphenol oxidases generates 3,4-dihydroxy-_L_-phenylalanine (_L_-DOPA) which is then accepted by TYDC that removes a carboxyl group from _L_-DOPA, forming dopamine (**Figure 4**) (Facchini and De Luca, 1994).

**Figure 5:**
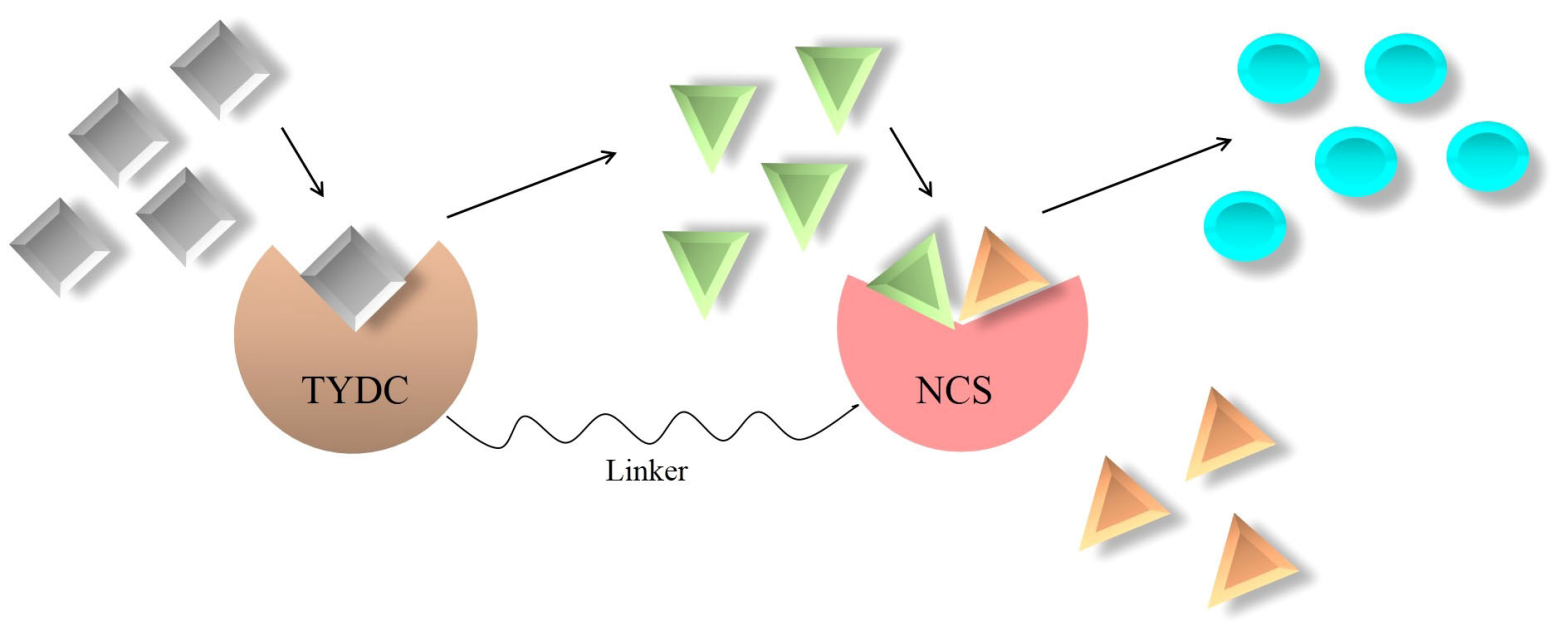
Artificially synthesized metabolon consisting of key enzymes involved in benzylisoquinoline alkaloid biosynthesis. NCS, norcoclaurine synthase; TYDC, _L_-tyrosine/_L_-DOPA decarboxylase; 
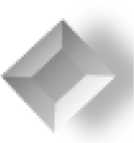
, _L_-DOPA; 
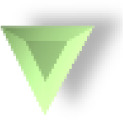
, dopamine; 
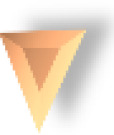
, 4-hydroxyphenylacetaldehyde; 
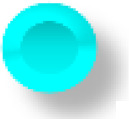
, (S)-norcoclaurine.

NCS displays cooperation between substrate-binding sites, meaning that the NCS-catalyzing step is rate limiting (Samanani and Facchini, 2001; Samanani and Facchini, 2002). Unfortunately, despite occupying the entry-point location and undertaking the regulatory task in BIA biosynthesis, NCS suffers from catalytic inefficiency. It is calculated that the average catalytic efficiency (*k*_cat_/*K*_m_) of enzymes is 125 mM, but this parameter for NCS plummets to 1.0 mM (Samanani and Facchini, 2001, 2002). A high *K*_m_ for both dopamine and 4HPAA highlights the fact that remarkable substrate concentrations should be afforded for NCS to achieve a reasonable turnover (*k*_cat_) (Lichman et al., 2015). Among the techniques employed to improve the enzyme kinetic parameters is the formation of a synthetic metabolon. In this regard, since NCS activity is substantially enhanced by an increase in dopamine content (Samanani et al., 2004), an enzyme (i.e., TYDC) that can provide dopamine for NCS is the best partner to fuse with NCS (**Figure 5**). Interestingly, TYDC shows a strong preference for dopamine than _L_-tyrosine (Facchini and De Luca, 1994). Thus, NCS–TYDC fusion is expected to supply more available dopamine for NCS and may increase its *k*_cat_ via confining produced dopamine to a small location and avoiding the entrance of it into competitive pathways, such as phenethylisoquinoline and emetine alkaloids (**Figure 5**) (Nomura and Kutchan, 2010; Polturak and Aharoni, 2018). This could lead to enhanced (*S*)-norcoclaurine amount and finally metabolic flux into BIA biosynthesis in opium poppy.

It is noteworthy that the direct fusion of proteins leaves the fusion enzymes crippled. They are unable to fold properly, perform biological activities, and generate sufficient products (Zhang et al., 2009). Therefore, choosing or designing short stretches of amino acids designated as linkers is of great importance. Linkers bring interacting proteins in close proximity, retain their bioactivity, and increase cooperative interactions (Gokhale and Khosla, 2000). Based upon amino acid residues, linkers are put into three groups: flexible, rigid, and cleavable. Flexible linkers usually consist of residues that are small in size and vary in polarity, such as Gly, Ser, and Thr (Chen et al., 2013). The proline-rich linkers possess a rigid structure bringing about hindrance to the mobility of and unfavorable interactions between joined proteins (Chen et al., 2013). Amino acid sequences susceptible to cleavage by enzymes, radiation, or chemicals make up cleavable linkers (Chen et al., 2013). The sequence of residues providing flexibility is among the most preferable linkers. They allow movement, improve conformation, and enhance the performance of the connecting enzymes (Chen et al., 2013). The most widely used flexible linker is (Gly_4_Ser)*n*, which can be inserted into the NCS and TYDC fusion (**Figure 5**). However, the number of repetition “*n*” must be optimized to acquire enzymes of proper folding and appropriate distance.

### 3.3 Targeting genes and miRNAs

Clustered regularly interspaced short palindromic repeats (CRISPR) have been around for approximately two decades in the biotechnology era. This recent methodology functions with the aid of a CRISPR-associated (Cas) endonuclease accompanied by a synthetic single guide RNA (sgRNA) with up to 30 nucleotides. The sgRNA tightly binds with the specific sequence within a gene, marking the desirable site for cleavage. The Cas9 nuclease enzyme recognizes the target location and heads the cleavage of the double-stranded DNA immediately, which is followed by a short sequence named protospacer-adjacent motif (PAM) (Collias and Beisel, 2021). The double-strand break triggers natural DNA repair mechanisms categorized into two distinct groups: non-homologous end joining (NHEJ) and homology-directed (HDR) (Arora and Narula, 2017). The repair mechanisms work well, but sometimes insertion/deletion (InDel) and substitution mutations occur in the target sequence(s). The discriminating attributes of CRISPR in contrast to conventional editing tools are five folds as: robustness, accuracy, sequence specificity, straightforwardness, and commercial effectiveness (Jaganathan et al., 2018). The capability of CRISPR for editing the BIA biosynthetic genes has been proven. The sgRNA was designed according to the *4*′*OMT2* gene, a regulatory gene acting prior to the divergence of different BIA pathways. The sgRNA-*4*′*OMT2* and Cas9 encoding vectors were delivered into the opium poppy leaves by means of *Agrobacterium.* The genetically transformed plant carried InDels. Accordingly, BIA production was markedly reduced (Alagoz et al., 2016).

**Figure 6:**
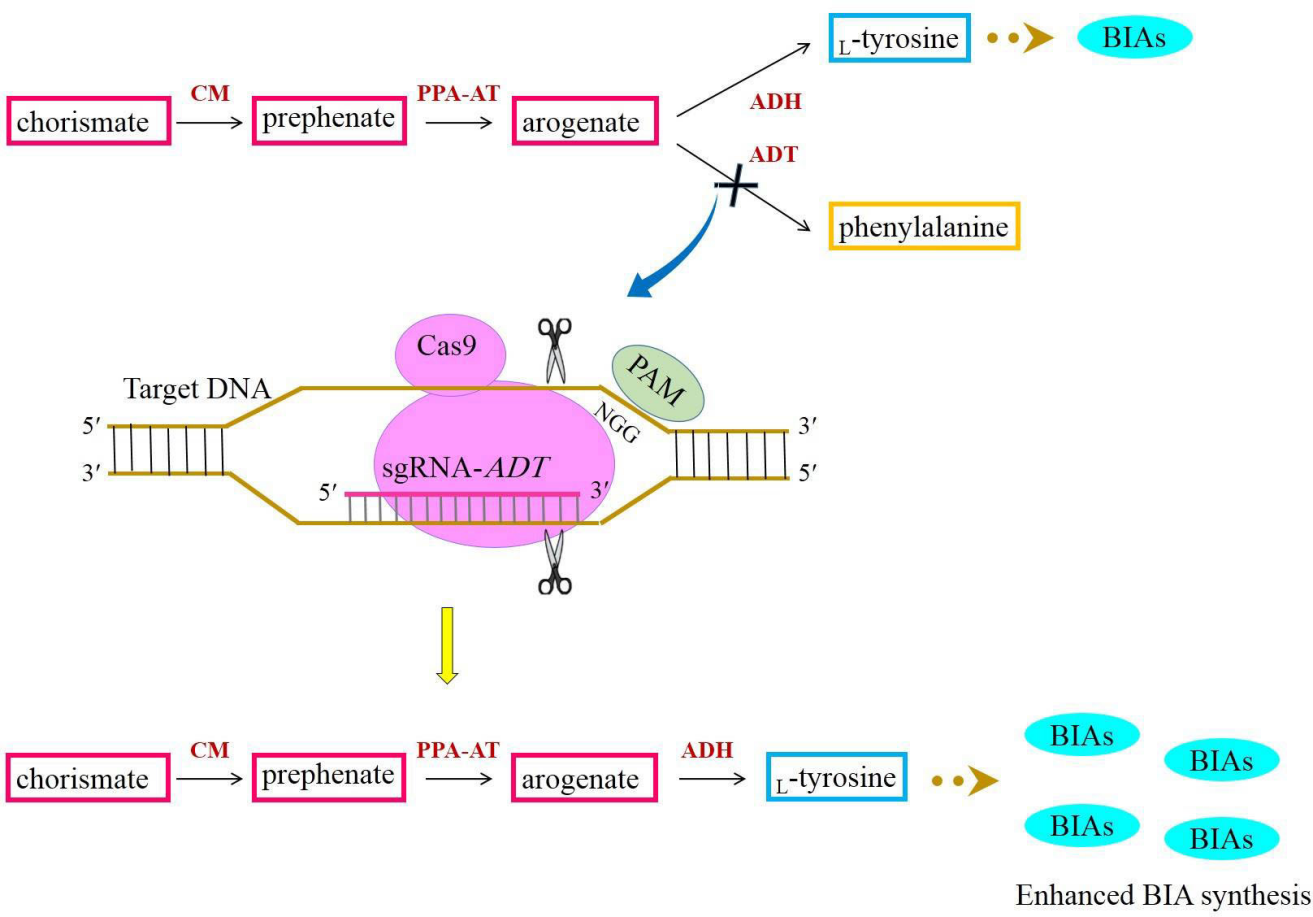
Acquiring higher BIA synthesis through directing more carbon from chorismate to _L_-tyrosine with the aid of the CRISPR/Cas9-mediated downregulation of *ADT*. ADH, arogenate dehydrogenase; ADT, arogenate dehydratase; BIAs, benzylisoquinoline alkaloids; CM, chorismate mutase; PAM, protospacer-adjacent motif; PPY-AT, phenylpyruvate aminotransferase.

The amino acid which donates the amine group to BIAs is _L_-tyrosine. _L_-Tyrosine is generated from the shikimate pathway responsible for the synthesis of myriad primary and secondary metabolites (Maeda and Dudareva, 2012). The steps involved in the shikimate pathway have been fully elucidated. In this pathway, chorismate is yielded via successive reactions. Chorismate is subsequently converted to prephenate by chorismate mutase (CM). The transamination of prephenate results in arogenate synthesis (Maeda and Dudareva, 2012). _L_-Tyrosine is a product of arogenate dehydration/decarboxylation catalyzed by arogenate dehydrogenase (ADH) (**Figure 6**). As a branch point intermediate, arogenate is also converted to phenylalanine by the activity of arogenate dehydratase (ADT) (Cho et al., 2007) (**Figure 6**). A growing body of evidence has indicated that the majority of carbon flux is channeled into phenylalanine biosynthesis (Maeda and Dudareva, 2012; Tohge et al., 2013). Hence, to allocate a high percentage of carbon to _L_-tyrosine biosynthesis, the suppression of *ADT* with the CRISPR/Cas9 system may direct more carbon to phenylalanine biosynthesis. If this happens, arogenate is highly likely to be converted to _L_-tyrosine frequently, culminating in plentiful BIAs (**Figure 6**).

Phenylalanine is a universal precursor utilized in the biosynthesis of an array of phenolic compounds, such as flavonoids, isoflavones, flavones, phenylpropanoids, anthocyanin, tannins, coumarin, lignans, and lignin (Tohge et al., 2013). Compounds stemmed from phenylalanine encompass 30% of organic matters in plant species (Tohge et al., 2013). It is notable that the plastids are predominantly cellular compartments bearing the phenylalanine biosynthetic route (Maeda and Dudareva, 2012; Lynch, 2022). However, the recent investigations conducted for determining whether the pathway is present in more than one subcellular localization have indicated that the pathway intermediates and enzymes converting chorismate to phenylalanine are localized in the cytosol alike (Lynch, 2022). It means that phenylalanine is also biosynthesized in the cytosol. Thus, if the plastidic *ADT* is knocked out, the poppy plants will not experience any deleterious effects resulting from the lack of phenylalanine and its derivatives. This is because the cytosolic phenylalanine pathway adequately supplies the plants with phenylalanine.

To globally enhance BIAs, other interesting targets for gene editing using CRISPR are microRNAs (miRNAs). miRNAs are small regulatory RNAs, with nearly 21 nucleotides. They are not capable of coding any proteins but post-transcriptionally regulate the expression of protein-coding genes. In fact, miRNAs connect with complementary sequences present in their target mRNAs, making transcripts vulnerable to cleavage by endonucleases and consequently hampering their translation (Brodersen et al., 2008). miRNAs involved in the growth and development, biotic and abiotic responses, signal transduction, cell differentiation and dedifferentiation, polarity, carbohydrate metabolism, protein conformation, compartmentalization, degradation, and gene transcription have been discovered and functionally characterized in opium poppy (Unver et al., 2010; Boke et al., 2015; Karimi et al., 2017). Furthermore, it has been documented that miRNAs can modulate BIA biosynthesis in poppy plants (Boke et al., 2015; Sabzehzari and Naghavi, 2019). The transcripts of 3′-hydroxy-*N*-methylcoclaurine 4′-*O*-methyltransferase (4′OMT) and 7-*O*-methyltransferase (7OMT) are subjected to pso-miR2161 and pso-miR13, respectively (Boke et al., 2015). The *4′OMT* and *7OMT* genes are responsible for the methylation of 3′-hydroxy-*N*-methylcoclaurine and (*S*)-reticuline, respectively (Morishige et al., 2000; Ounaroon et al., 2003). pso-miR408 likewise suppresses the genes encoding FAD-binding and BBE domain-containing protein (reticuline oxidase-like protein) (Boke et al., 2015) which form (*S*)-scoulerine from (*S*)-reticuline (Facchini et al., 1996). miRNAs targeting the mRNAs of codeinone reductase (COR), salutaridinol 7-*O*-acetyltransferase (SalAT), and TYDC have also been identified: pso-miR t0047847, pso-miR t0013376, and pso-miR t0000199, respectively (Boke et al., 2015). The two former enzymes yield codeine and thebaine, respectively (Unterlinner et al., 1999; Grothe et al., 2001), while the latter generates tyramine and dopamine (**Figure 4**) (Facchini and De Luca, 1994). The blocking of pso-miR t0000199 activity using CRISPR may allow the upregulation of *TYDC*, resulting in higher dopamine production. As depicted in **Figure 4**, dopamine is a precursor for NCS that synthesizes (*S*)-norcoclaurine, a distant BIA pathway intermediate. Therefore, by editing the pso-miR t0000199-encoding gene, the poppy plants may witness increased BIA biosynthesis and accumulation.

## 4 Conclusion and future perspectives

People of all ages are utilizing plant-derived medicines, in particular, pharmaceutical BIAs, such as morphine and codeine, to relieve their pains. Their expensive chemical synthesis was due largely to their complicated structures coupled with their sparse amounts in plants, which are highly likely to result in the dwindling shortage of herbal remedies. To combat this problem, enhancing the accumulation of such bioactive compounds in the plants that produce them appears to be the most feasible and effective strategy. In this regard, many endeavors have been made with notable successes. However, further studies are still required to identify plants with remarkable concentrations of bioactive compounds. The methods presented here can open a new horizon for further research. If accompanied by omics technologies, bioinformatics, and sequencing, the strategies reviewed and suggested here can broaden our knowledge about regulatory networks and help us to manipulate BIA pathways more precisely, leading to the achievement of the best results.

It is assumed that, in the near future, the techniques mentioned above will be used not only to produce BIAs but also to synthesize other medicinal compounds as they positively affect the metabolic profiles of the plants from which those metabolites originate. They, therefore, would live up to the pharmaceutical industry’s expectations.

## Author contributions

ZA: Writing – original draft, Visualization, Investigation, Conceptualization. MN: Writing – review & editing, Supervision. MZ: Writing – review & editing.
